# Variability of diabetic macular edema in correlation with hypertension retinopathy in patients with diabetes mellitus and essential hypertension

**Published:** 2019

**Authors:** Daniela Stana, Vasile Potop, Sînziana Luminiţa Istrate, Cecilia Eniceicu, Ana Raluca Mihalcea, Irena Gabriela Paşca, Abdallah Aqel, Radu Ciuluvică, Dana Moraru

**Affiliations:** * Ophthalmology Department, University Emergency Hospital, Bucharest, Romania; ** Ophthalmology Department, “Carol Davila” University of Medicine and Pharmacy, Bucharest, Romania; *** Anatomy Department, “Carol Davila” University of Medicine and Pharmacy, Bucharest, Romania; **** Academic Center for Optical Engineering and Photonics, Politehnica University, Bucharest, Romania

**Keywords:** diabetes, blood pressure, macula, contrast sensitivity

## Abstract

**Objective.** This study aimed to determine the possible relationship between variability of diabetic macular edema associated with hypertension retinopathy in patients with type 2 diabetes mellitus and essential high blood pressure, in correlation with contrast sensitivity.

**Material and Methods.** In order to accomplish the objective, this retrospective study evaluated the progression of diabetic macular edema in patients with high blood pressure during day time through measurements of the total macular volume and central macular thickness using optical coherence tomography and contrast sensitivity variations measured through Pelli Robson test, four times a day, along with glycemia and blood pressure measurements.

**Results.** Our results showed a statistically significant correlation between the values of glycemia and central macular thickness, and between contrast sensitivity and macular thickness at every tested hour.

**Conclusions.** The study revealed many statistically significant correlations involving blood pressure, blood glucose levels, Pelli Robson test and central macular thickness.

**Abbreviations:** OCT = optical coherence tomography; DME = diabetic macula edema; SD-OCT = spectral domain optical coherence tomography; LOCS = lens opacities classification system; LE = left eye; ANOVA = analysis of variance

## Introduction

Worldwide it has been found that diabetes mellitus is an essential cause of blindness and represents the leading cause of severe loss of vision in people of working age in many countries [**1**]. Over time, high blood glucose levels lead to the development of diabetic retinopathy and high blood pressure might increase the risk of rapid progression and earlier onset of the disease [**2**].

Optical coherence tomography (OCT) has made it possible to enlarge our knowlegde of diabetic retinopathy and to quantify the retinal edema.

Fenwick et al. demonstrated that poor glucose and blood pressure control leads to a higher risk of developing diabetic retinopathy and macular edema than poor glycemic control alone [**3**].

Sometimes, patients with poor blood pressure and glucose control may have a fulminant evolution of their diabetic retinopathy [**4**]. An important objective of this paper was to highlight the physiological mechanisms of the progression of macular edema in patients with type 2 diabetes mellitus and essential hypertension, in correlation with contrast sensitivity using paraclinical imaging methods (OCT).

Glycemic variations in a patient with diabetes mellitus suppose clinical changes, sometimes significant, like decreased visual acuity, changes of the ocular crystalline refractive index and decreased contrast sensitivity explained through the increased permeability of the capillaries in the macula. The elements of severity are brought by blood pressure variability and a fragile control of blood glucose levels during a day so that the macular edema progresses with deterioration of the parameters.

This retrospective study analyzed the progression of the diabetic macular edema in patients with high blood pressure due to a poor glycemic and systemic blood pressure control during a day in correlation with the decrease of contrast sensitivity through measurements of the total macular volume and the central macular thickness using the optical coherence tomography.

## Methods and materials

a) Study design: The present paper presented a cohort, prospective, non-randomized, comparative clinical study, which analyzed 45 patients hospitalized in the Ophthalmology Clinic of University Emergency Hospital in Bucharest with the diagnosis of diabetic retinopathy and type 2 diabetes mellitus, which was poor controlled. This study was undergone between March and June 2015. This study was conducted according to the Helsinki Convention regarding the patients’ rights. The informed consent was presented and signed by all the participants after the presentation of the study’s objectives.

b) Patients selection: the patients selected in this study have met the inclusion and exclusion criteria presented in **Table 1**.

**Table 1:** The inclusion and exclusion criteria of the patients enrolled in the study

Inclusion Criteria - time 9.00 AM	Exclusion Criteria-time 9.00 AM
Systemic Blood Pressure > 140/ 90 mm Hg	Systemic Blood Pressure < 140/ 90 mm Hg
Glycemia > 150 mg/ ml	Glycemia < 150 mg/ dl
Visual Acuity over 0.3 wpc	Visual Acuity under 0.3 wpc
DME at the examination with OCT	Absence of DME
The absence of the opacities of the ocular media	The presence of the opacities of the ocular media (cataract over second degree LOCS)

**c) Description of the group**

45 patients (in total 85 eyes) with type 2 diabetes, poor controlled and DME, who were sent by their diabetologist for the regular eye examination, were enrolled in this study. The patients were under supervision and examined through 4 tests for 24 hours: the initial examination at 9.00 am, at 12.00 pm, 3.00 pm and 6.00 pm through ophthalmic examination and a general clinical examination.

**d) General clinical evaluation**

The patients were generally clinically evaluated through the measurement of their vital parameters, pulse, systolic and diastolic blood pressure, respiratory frequencies, through two consecutive measurements, at 20 minutes distance from one another.

**e) Clinical evaluation** consisted in testing the glycemic level through blood samples, the samples were examined in the same day of the prelevation, by the same laboratory, with the same investigation apparatus, during the entire clinical study.

**f) Ophtalmologic evaluation**

All the patients were evaluated at 9.00 am through a complete ophthalmic examination, the data were analysed and quantified with the following parameters:

- Visual acuity;

- Pelli-Robson Test;

- Intraocular Pressure (IOP);

- Biomicroscopic examination of the anterior and posterior pole;

- Retinophotography examination; 

- The macular examination through the optical coherence tomography (OCT).

Visual acuity was checked with the Snellen chart and retinoscope, and the absence of visual acuity useful to the investigated eye was considered an exclusion criterion (over 0.3 ccp).

The Pelli-Robson test measured the sensitivity to contrast by detecting letters whose color intensity gradually decreased on a white background. The test was performed at 1 m under a 85 cd/ m2 luminance and detected a decrease in contrast sensitivity for ocular pathologies such as glaucoma, cataract, diabetic retinopathy. Patients with significant media opacities (cataracts over II LOCS) were excluded from the study because the sensitivity to contrast could have been significantly affected.

Intraocular pressure was measured in most patients by Goldmann aplanotonometry, those with high intraocular pressures were excluded due to the need for ocular hypotonizing.

Biomicroscopic examination of the anterior pole was performed for each patient. The indirect posterior pole examination was performed using a biomicroscope and a Volk Super Field lens. The presence of crystalline opacities (LOCS cataract) or corneal opacities was an exclusion criteria.

The retinophotographic examination of the eyeball was performed in all cases with the Fundus Camera Zeiss after the pupil’s dilation. The presence of diabetic macular edema was the main inclusion criteria.

Spectral field tomography (SD-OCT, 512 A-scan, 20 × 15) for the macular region was performed using the Zeiss Cirrus 4000 OCT after pupil dilation, with the macular volume and macular central thickness being measured.

**g) Data processing**

Within the study, statistical analysis and graphics were made with 20 SPSS (Statistical Package for the Social Sciences). SPSS is a modular line of fully integrated products for the analytical process-planning, data collection, access, training, data management and analysis in the bargain for reports and presentation of the results. Statistical data analysis was done on a sample composed of 45 people - 85 eyes. Due to their type and formulated objectives, the cross table and Chi-square test were used in the research, in addition to the descriptive analysis of the variables (mean, median, standard deviation, modal value, minimum and maximum) [**5**-**7**].

## Results

In the study, 24 females (53.33%) and 21 males (46.66%) were included. The average age was 72.8 years.

**Table:** **Confidence Intervals for Estimated**. **Mean of Population**.

For .95 CI:	72.8 ± 5.3539
For .99 CI:	72.8 ± 7.4554

**Fig. 1:**
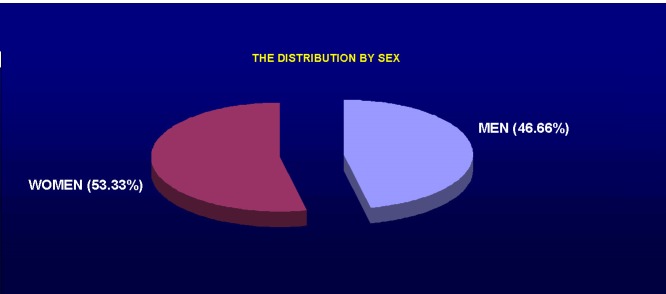
Sex distribution of patients

In terms of distribution by age groups, the majority of the patients, 14, 31.11%, was in the 70-79 years old group. In the group under the age of 50 years there were 2 patients, between 50-59 years old, 6 patients, between 60-69 years old, 12 patients, between 80-89 years old, 8 patients, and in the group over 90, 3 patients.

**Fig. 2:**
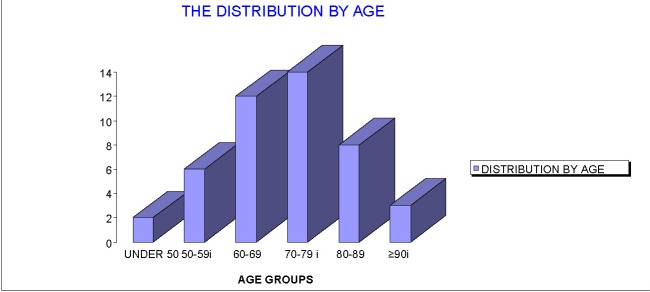
Age distributions of patients

A table was made for each patient in the study with the obtained data. In the following table, the data for the first 15 patients is presented in **Table 2**.

**Table 2:** The data for the first 15 patients

Patient	Sex	Age	Time	Blood pressure	Blood glucose	TEST P.R. / P.K. (%)
1	F	69	9:00	170/ 80	171	5
			12:00	180/ 90	186	5
			15:00	160/ 75	360	5
			18:00	130/ 80	242	5
2	M	68	9:00	155/ 90	147	25
			12:00	170/ 90	134	15
			15:00	160/ 70	211	15
			18:00	160/ 80	181	25
3	F	80	9:00	190/ 90	122	5
			12:00	165/ 80	181	3.5
			15:00	150/ 70	160	3.5
			18:00	170/ 80	146	5
4	F	59	9:00	150/ 90	170	15
			12:00	130/ 75	227	25
			15:00	170/ 90	218	15
			18:00	185/ 80	231	25
5	M	63	9:00	160/ 90	177	2
			12:00	140/ 90	193	3.5
			15:00	150/ 90	142	2
			18:00	165/ 80	158	2
6	M	68	9:00	150/ 85	164	5
			12:00	140/ 85	149	5
			15:00	120/ 70	242	5
			18:00	130/ 80	68	5
7	F	77	9:00	150/ 90	168	3.5
			12:00	140/ 90	100	5
			15:00	150/ 90	204	2
			18:00	165/ 80	111	5
8	F	82	9:00	150/ 90	150	5
			12:00	160/ 90	126	3.5
			15:00	130/ 70	106	5
			18:00	140/ 80	124	5
9	M	83	9:00	160/ 80	193	10
			12:00	150/ 90	174	10
			15:00	160/ 90	150	12
			18:00	165/ 90	180	10
10	F	54	9:00	170/ 80	184	10
			12:00	160/ 90	190	15
			15:00	160/ 90	176	12
			18:00	165/ 85	158	12
11	M	69	9:00	190/ 100	162	15
			12:00	180/ 90	170	12
			15:00	190/ 110	145	10
			18:00	185/ 100	153	15
12	F	86	9:00	160/ 90	211	5
			12:00	140/ 85	181	2
			15:00	170/ 90	122	5
			18:00	185/ 80	181	5
13	F	79	9:00	170/ 90	157	2
			12:00	180/ 90	134	3.5
			15:00	160/ 75	211	2
			18:00	130/ 80	181	2
14	M	71	9:00	180/ 90	180	15
			12:00	175/ 80	217	20
			15:00	150/ 70	210	15
			18:00	170/ 80	231	25
15	F	84	9:00	155/ 90	242	5
			12:00	180/ 90	98	5
			15:00	160/ 70	168	5
			18:00	160/ 80	100	5

In addition, for each eye that was included in the study, for each hour the measurements were made, macular thickness value in the central subfield was followed, in perifoveolar subfields (superior, inferior, nasal, temporal) and in parafoveolar subfields (superior, inferior, nasal, temporal), according to the example presented in **Table 3** (in this case, patient no. 3, LE).

**Table 3:** Measurements in perifoveolar and parafoveolar subfields

	9:00	12:00	15:00	18:00
CENTRAL	297	178	61	177
N. PARA	295	277	176	290
N. PERI	268	217	195	270
I. PARA	252	293	59	285
I. PERI	283	233	38	250
S. PARA	269	287	257	291
S. PERI	246	220	259	251
T. PARA	278	269	210	272
T. PERI	234	71	236	232

The measurments were made at indicated times, with a significant difference of 30 minutes maximum.

The thickening of the retina was defined as the difference between retinal thickness obtained at 9:00 am and the normal one, 202 microns. The relative difference was followed too, which was the proportion between retinal thickness at 15:00 pm and 9:00 am and retinal thickening. The relative and absolute mean change was calculated with a confidence interval of 95%, considering the correlation between the eyes of the subjects included in the study. The proportion of eyes with changes of retinal thickness over 25% was calculated between 9:00 am and 15:00 pm or between 9:00 am and 18:00 pm, and was considered to be statistically significant.

The average blood sugar levels at 9:00 am was 171.4444 (+/ -14.1814) mg/ dl, range of confidence 95%. At 12:00 pm, the mean value was 182.3543 mg/ dl, at 15:00 pm it was 175.3322 mg/ dl and at 18:00 pm it was 170.4333 mg/ dl.

**Fig. 3:**
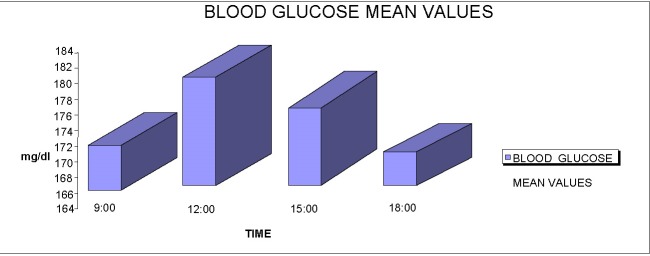
The average distribution of blood glucose level by hour

The mean blood pressure at 9:00 am was 158.4444 (+/ -5.1542) mm Hg. At 12:00 pm it was 163.2333 (+/ -6.1773) mm Hg, at 15:00 pm it was 161.3222 (+/ -5.8233) mm Hg and at 18:00 pm it was 160.0666 (+/ -5.9561) mm Hg.

**Fig. 4:**
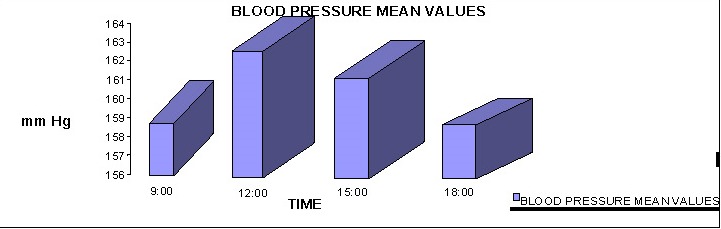
The average distribution of blood pressure by hour

There was a statistically significant difference (p = 0.044425) between the values of blood pressure and blood glucose at 9:00 am. This association was maintained at 15:00 am (p = 0.041275 and 0.042745 = 18:00 (p, but not at 12:00 pm).

**Fig. 5:**
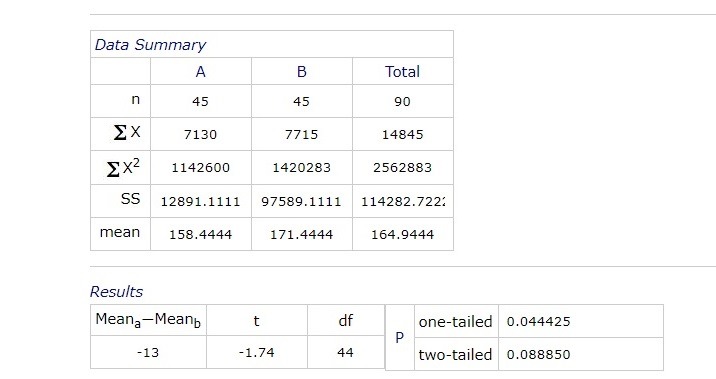
Correlation between blood pressure and blood sugar levels at 9:00 am

**Fig. 6:**
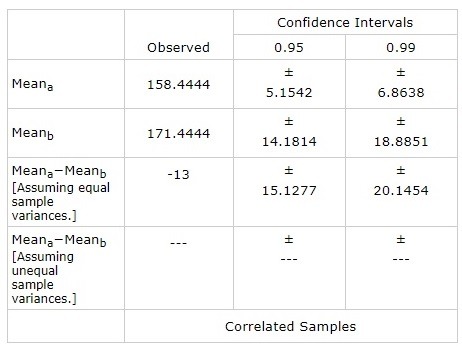
Correlation between blood pressure and blood sugar levels at 9:00 am

Comparatively, analyzing blood pressure and central macular thickness values at 9:00 am the following results were obtained:

**Fig. 7:**
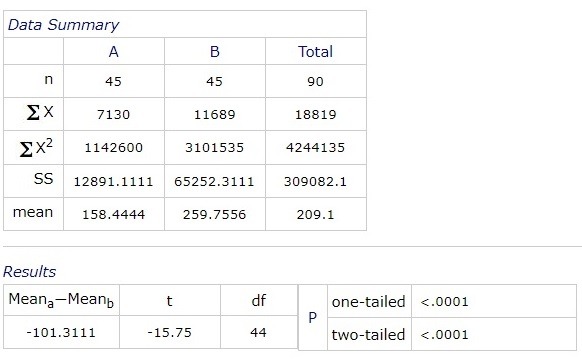
The value of blood pressure compared to the thickness of central macula at 9:00 am

A statistical significant difference between the values of blood glucose at 9:00 am and central macular thickness was obtained:

**Fig. 8:**
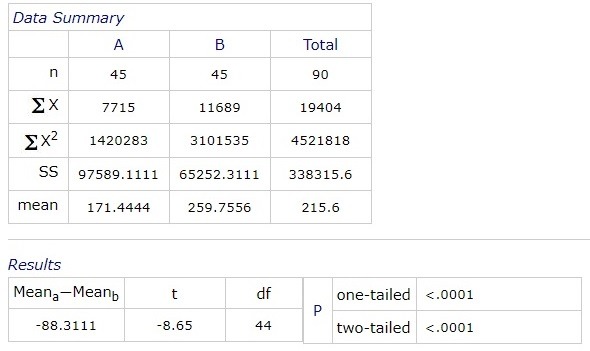
Correlation between blood pressure and blood sugar levels at 9:00 am

Next, we analyzed comparatively central macular thickness from 9:00 am, 12:00, 15:00 and 18:00 pm, in patients who met the conditions for entering the study. After performing ANOVA (analysis of variance), there were statistically significant differences between macular central thickness at 9:00 am and 15:00 am, and between 12:00 pm and 15:00 pm. There was no statistically significant difference between 9:00 am and 12:00 pm, and between 18:00 pm and other hours.

**Fig. 9:**
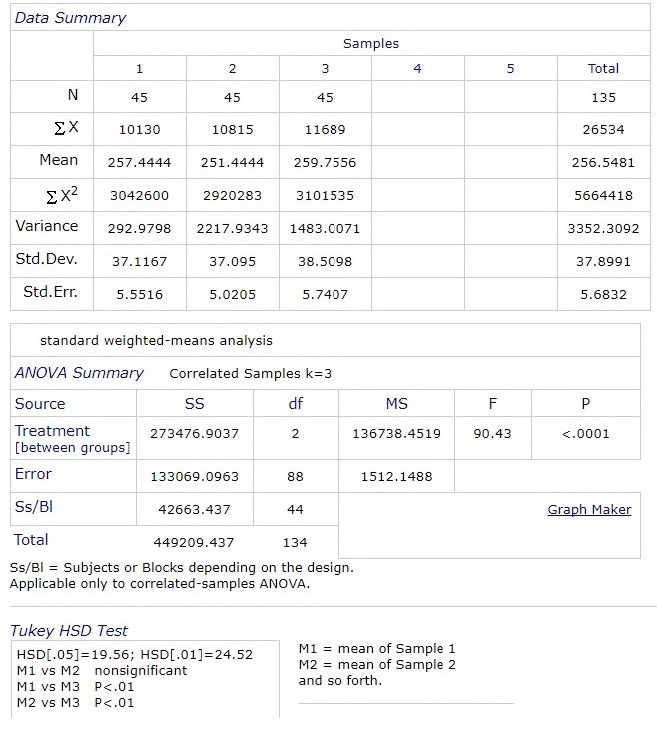
Comparative values of central macular thickness at 9:00 am, 12:00 pm and 15:00 pm

The correlation between contrast sensitivity (test Pelli-Robson) and macular thickness was analyzed at each of the given hours. A significant statistic correlation between the Pelli Robson test and central macular thickness was obtained, at every tested hour.

**Fig. 10:**
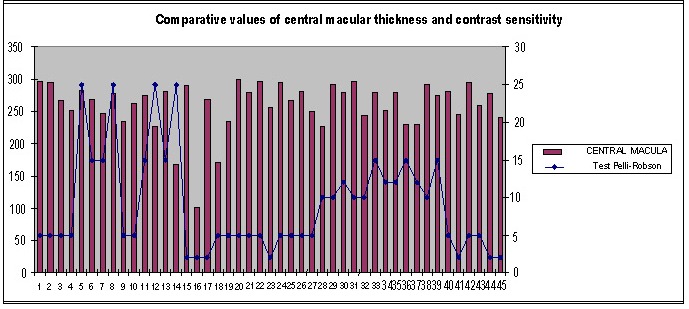
Comparative values of central macular thickness and contrast sensitivity at 9:00 am

**Fig. 11:**
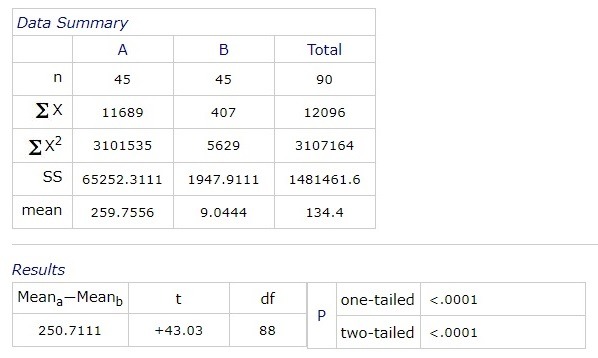
Comparative values of central macular thickness and contrast sensitivity at 9:00 am

## Conclusions

Average blood sugar levels at 9:00 am was 171.4444 (+/ -14.1814) mg/ dl, 95% interval of confidence. At 12:00 pm, the mean value was 182.3543 mg/ dl, at 15:00 pm it was 175.3322 mg/ dl and at 18:00 pm it was 170.4333 mg/ dl.

The average blood pressure at 9:00 am was 158.4444 (+/ - 5.1542) mm Hg. At 12:00 it was 163.2333 (+/ - 6.1773) mm Hg, at 15:00 it was 161.3222 (+/ - 5.8233) mm Hg and at 18:00 it was 160.0666 (+/ - 5.9561) mm Hg.

There was a statistically significant difference (p = 0.044425) between the blood pressure and blood glucose values at 9:00. This association was also maintained at 15:00 (p = 0.041275 and 18:00 (p = 0.042745), but not at 12:00.

There was a statistically significant difference (p <0.0001) between the glycemic value at 9:00 and the central macular thickness values. On a comparative analysis of the central macular thickness at 9:00, 12:00, 15:00 and 18:00, following the ANOVA test, there were statistically significant differences between the central macular thickness at 9:00 and 15:00, and between 12:00 and 15:00. There were no significant statistical differences between 9:00 and 12:00 and between 18:00 and the other hours.

A statistically significant correlation was obtained between the Pelli Robson test and the macular central thickness at each of the aforementioned time intervals.

**Disclosures**

None.

**Acknowledgment**

All authors have equal contribution to this paper.
